# Evaluation of serological changes of ESR, CRP, IL-6 and tumor necrosis factor-alpha (TNF-a) and hematologic parameters in pet dogs with pyometra

**DOI:** 10.1016/j.vas.2026.100922

**Published:** 2026-11-03

**Authors:** Hesamoddin Mohebbi, Mehrdad Neshat Gharamaleki, Bahram Amouoghli Tabrizi

**Affiliations:** Department of Clinical Sciences, Faculty of Veterinary Medicine, Tabriz Medical Science Branch, Islamic Azad University, Pasdaran Highway, Tabriz, East Azerbaijan, 5157944533, Iran

**Keywords:** Canine pyometra, C-reactive protein, Erythrocyte sedimentation rate, Interleukin-6, Tumor necrosis factor-alpha

## Abstract

Canine pyometra is associated with systemic inflammation, but erythrocyte sedimentation rate (ESR) has been less extensively evaluated alongside cytokines, C-reactive protein (CRP), and routine hematologic variables. This case-control study compared inflammatory and hematologic measurements in 15 dogs with closed pyometra and 15 clinically healthy controls. Interleukin-6 (IL-6), tumor necrosis factor-alpha (TNF-α), CRP, ESR, white blood cell (WBC) count, packed cell volume (PCV), and serum creatinine were assessed. Group differences were analyzed using independent-samples t-tests, with Welch correction when variance heterogeneity was detected. Mean differences were reported with 95% confidence intervals and Hedges' g effect sizes. Dogs with closed pyometra had higher IL-6, TNF-α, CRP, ESR, and WBC counts and lower PCV and serum creatinine than controls (all p ≤ 0.005). The largest standardized differences were observed for ESR (g = 2.39), PCV (g = −2.29), and serum creatinine (g = −1.94). Within the pyometra group, IL-6, TNF-α, and CRP were strongly correlated (r = 0.974–0.978; p < 0.001). These findings demonstrate a pronounced systemic inflammatory and hematologic phenotype in closed canine pyometra and identify ESR as a potentially informative component of multimarker inflammatory assessment. Prospective studies incorporating longitudinal measurements and clinical outcomes are required to determine diagnostic or prognostic utility.

## Highlights

• Closed pyometra was associated with marked systemic inflammatory changes.
• Erythrocyte sedimentation rate was higher in dogs with closed pyometra.
• White blood cell counts were higher and packed cell volume was lower.
• IL-6, TNF-α, and CRP were strongly correlated within pyometra cases.

## 1 Introduction

Pyometra is a common and potentially life-threatening uterine disease of intact female dogs that develops predominantly during diestrus and is characterized by bacterial infection, accumulation of inflammatory exudate within the uterus, and variable systemic inflammatory involvement. Severe disease may progress to systemic inflammatory response syndrome (SIRS), sepsis, endotoxemia, and organ dysfunction (Hagman, 2018; Smith, 2006; Verstegen et al., 2008). Diagnosis relies on reproductive and medical history, compatible clinical findings, laboratory abnormalities, and diagnostic imaging, particularly abdominal ultrasonography (Hagman, 2018; Jitpean et al., 2014).

Systemic inflammation in canine pyometra is accompanied by alterations in leukocyte counts, acute-phase proteins, inflammatory cytokines, and hematologic indices. CRP has been among the most consistently evaluated acute-phase proteins, whereas IL-6 and TNF-α are important components of the cytokine response (Fransson et al., 2007; Karlsson et al., 2012). More recent studies have demonstrated increases in CRP and leukocyte indices, changes in circulating inflammatory mediators, and dynamic biomarker responses after ovariohysterectomy (Ahn et al., 2021; Ceron et al., 2023; Goggs et al., 2022; Janković et al., 2022; Soler et al., 2021). A systematic review and meta-analysis further confirmed that leukocytosis and reductions in erythrocyte-related variables are recurrent findings in canine pyometra, whereas several biochemical measurements show substantial between-study heterogeneity (Demeli & Meyer, 2023).

The inflammatory phenotype may be particularly relevant in closed-cervix pyometra because impaired uterine drainage can be associated with marked uterine distension and systemic illness. Molecular evidence supports an intensified inflammatory response in closed-cervix disease: Özkan et al. (2024) reported approximately 10-fold upregulation of uterine TNF and IL6 expression in dogs with closed-cervix pyometra. Nevertheless, most clinical biomarker studies have focused on CRP, cytokines, leukocyte indices, or other emerging markers, whereas ESR remains comparatively underexplored in canine pyometra. ESR reflects erythrocyte aggregation influenced by plasma proteins and inflammatory changes and is widely used as a nonspecific inflammatory marker. Recent canine evidence has demonstrated higher ESR values in animals with inflammatory disease than in healthy animals and an association between elevated ESR and adverse outcomes in dogs with systemic inflammation (Joo et al., 2026).

The principal contribution of the present study is therefore not the first evaluation of CRP, IL-6, TNF-α, or conventional hematologic variables in canine pyometra, but their simultaneous assessment together with ESR specifically in dogs with closed pyometra. Evaluating these variables within the same population may provide a more integrated characterization of the systemic inflammatory phenotype and establish a rationale for future investigation of ESR as a complementary biomarker. The objective of this study was to compare ESR, CRP, IL-6, TNF-α, WBC count, PCV, and serum creatinine between dogs with closed pyometra and clinically healthy controls and to examine relationships among these variables within affected dogs. We hypothesized that closed pyometra would be associated with higher inflammatory-marker concentrations, ESR, and WBC counts together with measurable hematologic alterations.

## 2 Materials and methods

### 2.1 Study design and population

This observational case-control study included 30 client-owned intact female dogs: 15 dogs diagnosed with closed pyometra and 15 clinically healthy controls. Biological sex was female for all animals; gender was not applicable to this animal study. The mean age of the full study population was 5.2 ± 2.8 years (range, 2–10 years), and mean body weight was 12.5 ± 6.5 kg (range, 3–25 kg). The population included mixed-breed dogs, Terriers, Pomeranians, Miniature Pinschers, and other breeds.

Dogs with pyometra were recruited during 2023 from veterinary clinics and hospitals in Tabriz, Rasht, and Lahijan, Iran. Control dogs were recruited between September and October 2023 during routine clinical visits in Tabriz. The retained study records did not permit independent confirmation that enrollment was strictly consecutive; therefore, recruitment is described as clinic-based enrollment rather than consecutive sampling.

Group-specific distributions of age, body weight, and breed were not available and are therefore reported only for the study population as a whole. All animals were intact females, but detailed reproductive-cycle-stage information was not available from the retained study records. No a priori sample-size or power calculation was performed.

### 2.2 Diagnostic and eligibility criteria

Closed pyometra was diagnosed on the basis of compatible reproductive and medical history, clinical signs and physical examination findings, absence of purulent vaginal discharge consistent with a closed cervix, and abdominal ultrasonographic evidence of uterine fluid accumulation and distension of the uterine horns. This diagnostic approach is consistent with established clinical descriptions of canine pyometra (Hagman, 2018; Jitpean et al., 2014).Dogs with major concurrent systemic diseases capable of independently producing substantial inflammatory alterations were excluded. Dogs with documented recent administration of systemic antibiotics or anti-inflammatory drugs before initial blood sampling were also excluded when reliable medication histories were available. Blood sampling was performed before initiation of fluid therapy, antibiotics, anti-inflammatory medication, or surgical treatment.

Control dogs were presented for routine examination or vaccination and were considered clinically healthy on the basis of medical history, physical examination, absence of signs suggestive of systemic or reproductive disease, normal ultrasonographic appearance of the uterus, and available hematologic and biochemical findings. Similar clinically healthy female comparison populations have been used in contemporary canine pyometra biomarker studies (Ahn et al., 2021; Ceron et al., 2023).Anorexia was the most frequently documented presenting clinical sign among dogs with pyometra. The characteristic ultrasonographic pattern consisted of echogenic intrauterine fluid accumulation accompanied by dilation of the uterine horns.

### 2.3 Blood collection and sample processing

Approximately 5 mL of venous blood was collected aseptically from the cephalic vein at presentation and before any therapeutic or surgical intervention. Blood intended for complete blood count and ESR measurement was transferred to tubes containing dipotassium ethylenediaminetetraacetic acid (K₂EDTA). Blood intended for serum assays was collected into tubes without anticoagulant.

Serum samples were allowed to clot for approximately 30 minutes at room temperature and were subsequently centrifuged at approximately 1500 × g for 15 minutes. Separated serum was aliquoted and stored at −80°C until laboratory analysis. Samples were thawed only once before assay performance.

### 2.4 Laboratory measurements

Complete blood count measurements, including WBC count and PCV, were performed using a Mindray BC-2800Vet automated veterinary hematology analyzer.ESR was determined using the Westergren method and expressed in mm/h.

Serum CRP concentration was determined using an ALPCO canine CRP ELISA (catalog no. 41-CRPCA-E01). TNF-α and IL-6 concentrations were determined using R&D Systems canine DuoSet assays, catalog nos. DY1507 and DY1609, respectively, according to the manufacturers' procedures. Lot-specific analytical sensitivity, intra-assay and inter-assay coefficients of variation, and assay-run quality-control records were not available for the assay runs used in this study; values from unrelated assay lots were therefore not substituted.Serum creatinine concentration was measured using the kinetic Jaffe method on an automated biochemistry analyzer. For SI reporting, creatinine values originally expressed in mg/dL were converted to μmol/L using the conversion factor 1 mg/dL = 88.4 μmol/L.

### 2.5 Statistical analysis

Statistical analyses were performed using IBM SPSS Statistics version 26. Continuous variables are presented as mean ± standard deviation (SD). The original statistical workflow documented assessment of normality using the Shapiro-Wilk test. Individual Shapiro-Wilk test statistics and p-values were not retained and therefore cannot be re-reported.Between-group comparisons were performed using independent-samples t-tests. Homogeneity of variance was assessed using Levene's test. When the assumption of equal variance was satisfied, the conventional independent-samples t-test was reported; when variance heterogeneity was identified, Welch's unequal-variance t-test was used. The reported parametric comparisons therefore reflect the original statistical analysis. Because individual-level raw observations were not retained in a form permitting re-analysis, additional distributional diagnostics or nonparametric sensitivity analyses could not be independently reconstructed.

For each between-group comparison, the mean difference and corresponding 95% confidence interval (CI) were calculated. Standardized effect sizes were expressed as Hedges' g using the pooled standard deviation with correction for small-sample bias. Positive Hedges' g values indicate higher measurements in dogs with pyometra, whereas negative values indicate lower measurements.Within the pyometra group, relationships among PCV, TNF-α, IL-6, CRP, ESR, WBC count, and serum creatinine were evaluated using Pearson's correlation coefficient (r). Two-sided p-values are reported for all correlations. Because these analyses were exploratory, no adjustment for multiple comparisons was applied. Statistical significance was defined a priori as *p* < 0.05.

## 3 Results

### 3.1 Study population

Thirty intact female dogs were evaluated, including 15 dogs with closed pyometra and 15 clinically healthy controls. The available demographic, recruitment, reproductive, and clinical characteristics are summarized in Table 1.

**Table 1:** Study population, recruitment, and available clinical characteristics.

Characteristic	Description
Total study population	30 intact female dogs
Closed-pyometra group	n = 15
Healthy-control group	n = 15
Biological sex	Female
Reproductive status	Intact; detailed cycle-stage data unavailable
Age, overall cohort	5.2 ± 2.8 years (range, 2–10)
Body weight, overall cohort	12.5 ± 6.5 kg (range, 3–25)
Breeds represented	Mixed-breed, Terrier, Pomeranian, Miniature Pinscher, and other breeds
Pyometra recruitment	2023; Tabriz, Rasht, and Lahijan, Iran
Control recruitment	September–October 2023; Tabriz, Iran
Sampling documentation	Clinic-based enrollment; consecutive recruitment could not be independently confirmed
Principal presenting clinical sign	Anorexia
Principal ultrasonographic pattern	Echogenic uterine fluid accumulation with uterine-horn dilation

Note: Age, body-weight, and breed summaries represent the overall population because group-specific demographic distributions were unavailable.

### 3.2 Group comparisons

Dogs with closed pyometra had significantly higher IL-6, TNF-α, CRP, ESR, and WBC values than clinically healthy controls, whereas PCV and serum creatinine were significantly lower. Complete between-group comparisons are provided in Table 2.

**Table 2:** Comparison of inflammatory, hematologic, and biochemical variables between dogs with closed pyometra and healthy controls.

Variable	Pyometra group, mean ± SD	Control group, mean ± SD	Mean difference (95% CI)	Test statistic	p-value	Hedges' g
PCV (%)	28.82 ± 8.97	45.07 ± 3.90	−16.25 (−21.42 to −11.08)	t(28) = −6.438	<0.001	−2.29
TNF-α (pg/mL)	21.43 ± 11.82	11.37 ± 1.34	10.06 (3.49 to 16.63)	Welch t(14.36) = 3.276	0.005	1.16
IL-6 (pg/mL)	1943.94 ± 968.10	810.63 ± 294.97	1133.31 (580.93 to 1685.70)	Welch t(16.58) = 4.337	<0.001	1.54
CRP (mg/L)	88.65 ± 43.89	47.05 ± 18.16	41.60 (15.90 to 67.30)	Welch t(18.66) = 3.392	0.003	1.21
ESR (mm/h)	40.27 ± 18.03	8.87 ± 0.83	31.40 (21.41 to 41.39)	Welch t(14.06) = 6.736	<0.001	2.39
WBC (× 10⁹/L)	32.46 ± 23.75	11.26 ± 2.32	21.20 (8.01 to 34.39)	Welch t(14.27) = 3.441	0.004	1.22
Serum creatinine (μmol/L)	73.4 ± 16.8	107.8 ± 18.6	−34.5 (−47.7 to −22.1)	t(28) = −5.468	<0.001	−1.94

Note: n = 15 per group. CI, confidence interval; CRP, C-reactive protein; ESR, erythrocyte sedimentation rate; IL-6, interleukin-6; PCV, packed cell volume; SD, standard deviation; TNF-α, tumor necrosis factor-alpha; WBC, white blood cell count. Serum creatinine values were converted from mg/dL to μmol/L using a factor of 88.4. Conversion of units does not alter test statistics, p-values, or standardized effect sizes.

Relative to the control group, mean ESR was approximately 4.5-fold higher, WBC count 2.9-fold higher, IL-6 2.4-fold higher, and TNF-α and CRP approximately 1.9-fold higher in dogs with closed pyometra. Mean PCV was approximately 36% lower and serum creatinine approximately 32% lower.

### 3.3 Correlations within the pyometra group

Pairwise correlations among measured variables are presented in Table 3. Strong positive correlations were observed between TNF-α and IL-6 (r = 0.974, *p* < 0.001), TNF-α and CRP (r = 0.978, *p* < 0.001), and IL-6 and CRP (r = 0.977, *p* < 0.001). The correlation between ESR and serum creatinine was r = 0.514 with a reported *p* = 0.050 and was therefore not interpreted as statistically significant under the predefined criterion of *p* < 0.05.

**Table 3:** Pearson correlations among measured variables in dogs with closed pyometra.

Variable pair	r	p-value
PCV – TNF-α	0.058	0.839
PCV – IL-6	−0.043	0.878
PCV – CRP	−0.088	0.754
PCV – ESR	−0.002	0.995
PCV – WBC	−0.005	0.987
PCV – serum creatinine	0.245	0.379
TNF-α – IL-6	0.974	<0.001
TNF-α – CRP	0.978	<0.001
TNF-α – ESR	−0.200	0.474
TNF-α – WBC	−0.124	0.659
TNF-α – serum creatinine	−0.123	0.661
IL-6 – CRP	0.977	<0.001
IL-6 – ESR	−0.211	0.450
IL-6 – WBC	−0.135	0.632
IL-6 – serum creatinine	−0.069	0.808
CRP – ESR	−0.159	0.572
CRP – WBC	−0.116	0.681
CRP – serum creatinine	−0.119	0.673
ESR – WBC	0.399	0.141
ESR – serum creatinine	0.514	0.050
WBC – serum creatinine	0.455	0.088

Note: n = 15. Pearson's r and corresponding two-sided p-values are reported. CRP, C-reactive protein; ESR, erythrocyte sedimentation rate; IL-6, interleukin-6; PCV, packed cell volume; TNF-α, tumor necrosis factor-alpha; WBC, white blood cell count.

## 4 Discussion

The present study demonstrates a coordinated systemic inflammatory and hematologic phenotype in dogs with closed pyometra. Affected dogs showed higher ESR, CRP, IL-6, TNF-α, and WBC counts and lower PCV than healthy controls. Among the positive differences, ESR had the largest standardized effect size (Hedges' g = 2.39). The simultaneous assessment of these measurements is the principal contribution of the study; the findings do not imply that the individual biomarkers themselves are novel or that any of them has established diagnostic or prognostic performance.

Leukocytosis was prominent in the affected dogs, with a mean WBC count of 32.46 × 10⁹/L compared with 11.26 × 10⁹/L in controls. Ahn et al. (2021), studying 90 dogs with pyometra and 26 healthy female dogs, reported WBC values of 24.29 and 10.33 × 10⁹/L, respectively. Zheng et al. (2022) reported 19.25 ± 12.70 versus 9.61 ± 2.00 × 10⁹/L. In a meta-analysis of 44 studies, Demeli and Meyer (2023) estimated a pooled WBC count of 27.75 × 10⁹/L in pyometra and a mean difference of 17.16 × 10⁹/L (95% CI, 14.85–19.47) relative to controls. Domrazek et al. (2026) reported median WBC counts of 19.87 × 10⁹/L in closed-cervix pyometra, 21.43 × 10⁹/L in open-cervix pyometra, and 7.35 × 10⁹/L in controls. Differences among studies may reflect disease severity, timing of sampling, population characteristics, and data distribution rather than cervical status alone.

PCV was also markedly lower in affected dogs, with a mean difference of −16.25 percentage points and Hedges' g = −2.29. Ahn et al. (2021) reported hematocrit values of approximately 39% in dogs with pyometra and 47% in healthy dogs. Ceron et al. (2023) described mild normocytic normochromic non-regenerative anemia in five of 25 affected dogs, with hematocrit values of 30–35%. Domrazek et al. (2026) reported median hematocrit values of 43% in dogs with closed-cervix pyometra and 45% in controls. The lower PCV observed in the present population indicates a more pronounced erythrocyte-related alteration; however, inflammation, hydration status, disease duration, and other unmeasured factors may have contributed, consistent with the between-study heterogeneity described by Demeli and Meyer (2023).

CRP was approximately 1.9-fold higher in affected dogs (88.65 ± 43.89 mg/L) than in controls (47.05 ± 18.16 mg/L), with Hedges' g = 1.21. Soler et al. (2021) reported a mean CRP concentration of 147 ± 91 mg/L in dogs with pyometra, whereas Zheng et al. (2022) reported 22.20 ± 11.22 mg/L in affected dogs and 4.33 ± 2.34 mg/L in controls. Absolute concentrations should not be considered directly interchangeable among studies because assay platform, calibration, sample handling, case mix, and inflammatory severity may differ. Nevertheless, the direction of change is consistent with other studies reporting increased or persistently elevated CRP in canine pyometra (Ahn et al., 2021; Ceron et al., 2023; Janković et al., 2022).

IL-6 was approximately 2.4-fold higher and TNF-α approximately 1.9-fold higher in dogs with closed pyometra than in controls. Domrazek et al. (2026) reported a median peripheral-blood IL-6 concentration of 118.2 pg/mL in dogs with closed-cervix pyometra compared with 0 pg/mL (range, 0–5.19 pg/mL) in controls. In contrast, Ahn et al. (2021) did not detect a significant baseline IL-6 difference in a population consisting predominantly of dogs with open pyometra, although IL-6 changed after ovariohysterectomy. Variation in cytokine half-life, disease stage, timing of sampling, and immunoassay methodology may contribute to these differences. At the tissue level, Özkan et al. (2024) reported approximately 10-fold upregulation of TNF and IL6 expression and fivefold upregulation of IL10 in closed-cervix pyometra. Tissue gene expression and circulating protein concentrations are not directly equivalent, but both observations support substantial inflammatory activation in closed-cervix disease.

ESR was approximately 4.5-fold higher in affected dogs and had the largest positive standardized difference among the variables evaluated. Because ESR remains comparatively underexplored in canine pyometra, this finding is of particular interest. Joo et al. (2026), in a study of 350 dogs, reported median ESR values of 13 mm/h (IQR, 10–18) in diseased dogs and 10 mm/h (IQR, 4–11) in healthy dogs. They identified 12 mm/h as a threshold for discrimination between diseased and healthy dogs and reported an association between ESR >18 mm/h and mortality among dogs meeting SIRS criteria. The substantially higher mean ESR observed in the present pyometra population cannot be interpreted using those prognostic thresholds because the populations, disease spectrum, sampling, and outcomes differed. Rather, the finding supports prospective evaluation of ESR as a complementary component of inflammatory assessment in pyometra.

The correlations among IL-6, TNF-α, and CRP were exceptionally strong (r = 0.974–0.978; all *p* < 0.001). Özkan et al. (2024) similarly demonstrated relationships among inflammatory pathways in uterine tissue, and Ahn et al. (2021) reported associations between CRP and clinical variables related to inflammation and organ injury. However, correlations approaching unity in only 15 affected dogs require particular caution. The present analyses were exploratory, involved multiple pairwise tests without multiplicity adjustment, and cannot establish causal coupling, common regulation, or synchronized biomarker kinetics.

Serum creatinine was lower in affected dogs (73.4 ± 16.8 μmol/L; equivalent to 0.83 ± 0.19 mg/dL) than in controls (107.8 ± 18.6 μmol/L; equivalent to 1.22 ± 0.21 mg/dL). This contrasts with Domrazek et al. (2026), who reported median creatinine concentrations of 1.7 mg/dL in both open- and closed-cervix pyometra and 0.95 mg/dL in controls. Demeli and Meyer (2023) also documented substantial heterogeneity in serum biochemical changes across pyometra studies. Because creatinine is influenced by renal filtration, muscle mass, hydration, nutritional condition, and pre-analytical factors, the lower value in the present study should be regarded as a cohort-specific association rather than evidence of a characteristic renal response to closed pyometra.

The single-time-point design is important when interpreting these inflammatory measurements. Goggs et al. (2022) demonstrated time-dependent CRP changes in dogs with pneumonia, septic peritonitis, and pyometra, with a median of approximately 7 days for CRP to decline to 20% of its peak value. Ahn et al. (2021) demonstrated postoperative changes in inflammatory variables, and Janković et al. (2022) documented dynamic CRP and hematologic changes during the first 72 hours after surgery. The current measurements therefore characterize the inflammatory state at presentation but cannot define biomarker trajectories during treatment or recovery.

The potential value of multimarker assessment is also consistent with previous work examining integrated hematologic indices. Yazlık et al. (2022) reported areas under the receiver operating characteristic curve of 0.936 and 0.966 for the neutrophil-to-lymphocyte and monocyte-to-lymphocyte ratios, respectively, for identifying SIRS in dogs with pyometra and 0.955 and 0.931 for discrimination of pyometra. Güneş et al. (2025) similarly reported increases in CRP and WBC counts, changes in inflammatory cell ratios, and alterations in albumin and trace-element profiles. These findings reinforce the concept of integrated inflammatory phenotyping; however, the present study did not evaluate ROC curves, sensitivity, specificity, likelihood ratios, independent incremental value, or clinical decision thresholds and therefore cannot establish diagnostic performance for ESR or any multimarker combination.

Several limitations should be considered. The sample size was small, with 15 dogs per group, and no a priori sample-size calculation was performed, limiting statistical precision and generalizability. Enrollment was clinic based, and consecutive recruitment could not be independently confirmed from the retained records. Cases were recruited from three cities whereas controls were recruited in Tabriz, introducing the possibility of center- or location-related selection effects. Group-specific age, body-weight, and breed distributions and detailed reproductive-cycle-stage data were unavailable, limiting assessment of potential demographic and reproductive confounding. The case-control and single-time-point design precluded evaluation of longitudinal biomarker changes. Disease-severity scores, bacterial culture results, hospitalization duration, postoperative complications, treatment response, and survival outcomes were unavailable. Lot-specific ELISA analytical sensitivity, precision, and quality-control records were also unavailable. Individual Shapiro-Wilk outputs and individual-level raw observations were not retained in a form that allowed independent re-analysis, additional distributional assessment, identification of influential observations, nonparametric sensitivity analysis, or construction of valid individual-data plots. Finally, the correlation analyses were exploratory and were not adjusted for multiple comparisons. These limitations preclude validation of diagnostic, prognostic, or monitoring performance.

Despite these limitations, the coherent elevation of ESR, CRP, IL-6, TNF-α, and WBC count together with the reduction in PCV demonstrates substantial systemic inflammatory and hematologic alteration in dogs with closed pyometra. ESR may complement, rather than replace, established inflammatory measurements. Larger prospective multicenter studies should combine serial ESR measurements with standardized CRP and cytokine assays, detailed demographic and reproductive characterization, bacterial culture, disease-severity classification, and clinically relevant outcomes to determine whether ESR provides independent diagnostic, prognostic, or monitoring information.

## 5 Conclusions

Dogs with closed pyometra showed marked systemic inflammatory and hematologic alterations characterized by higher IL-6, TNF-α, CRP, ESR, and WBC counts and lower PCV and serum creatinine than healthy controls. ESR demonstrated the largest positive standardized difference between groups, whereas IL-6, TNF-α, and CRP were strongly correlated within affected dogs. These results identify ESR as a potentially informative component of the inflammatory profile of closed canine pyometra but do not establish diagnostic, prognostic, or monitoring performance. Larger prospective studies incorporating serial biomarker measurements, standardized assay validation, detailed clinical phenotyping, and clinically relevant outcomes are required.

## Ethics declaration

This study was conducted in accordance with the ARRIVE (Animal Research: Reporting of In Vivo Experiments) guidelines. This study was approved by the Research Ethics committees of Islamic Azad university-Tabriz branch.

## Declaration of generative AI and AI-assisted technologies in the manuscript preparation process

During the preparation of this work, the authors used OpenAI ChatGPT to assist with language editing. After using this tool, the authors reviewed and edited the content as needed, independently verified the scientific content and cited sources, and take full responsibility for the content of the published article.

## Ethics approval and consent to participate

All experimental protocols, animal-handling procedures, and sample collections were reviewed and approved by the Ethics Committee of Tabriz Medical Science Branch, Islamic Azad University, Tabriz, Iran (Ethics Code: IR.IAU.TABRIZ.REC.1402.015). Informed consent was obtained from all pet owners or legal guardians before inclusion of their dogs in the study and before clinical examination and blood sample collection.

## Consent for publication

Not applicable.

## Data availability

Individual-level raw observations from the original study were not retained in a form that permits independent re-analysis. The retained aggregate statistical outputs and study documentation supporting the reported results are available from the corresponding author upon reasonable request, subject to applicable ethical and institutional requirements.

## CRediT authorship contribution statement

**Hesamoddin Mohebbi:** Resources, Project administration, Investigation, Writing – original draft, Writing – review & editing. **Mehrdad Neshat Gharamaleki:** Writing – review & editing, Writing – original draft, Validation, Supervision, Conceptualization, Methodology. **Bahram Amouoghli Tabrizi:** Writing – review & editing, Writing – original draft, Validation, Supervision, Methodology, Data curation.

## Declaration of competing interest

The authors declare that they have no known competing financial interests or personal relationships that could have appeared to influence the work reported in this paper.
